# Transesophageal echocardiography, more than a diagnostic tool: use during surgical ligation of coronary artery fistulae - a case report

**DOI:** 10.1186/1749-8090-7-28

**Published:** 2012-04-06

**Authors:** Ping-Chen Chung, Pai-Ching Huang, Yu-Fang Liu, Kuen-Bao Chen

**Affiliations:** 1 Department of Anesthesia, Pain Service, and Critical Care Medicine, China Medical University Hospital, No. 2, Yuh-Der Road, Taichung 404, Taiwan, R.O.C; 2 College of Medicine, China Medical University, No.91 Hsueh-Shih Road, Taichung, Taiwan 404, Taiwan, R.O.C

**Keywords:** Coronary artery fistulae (CAF), intraoperative, transesophageal echocardiography (TEE)

## Abstract

Coronary artery fistulae (CAF) are an infrequent coronary abnormality. Herein, we describe the use of intraoperative transesophageal echocardiography (TEE) in the treatment of CAF. A 61 year-old woman presented with chest pain and symptoms consistent with unstable angina. Subsequent coronary angiography revealed the presence of 2 CAF, one extending from the left anterior descending artery to the pulmonary artery (PA) and the other extending from the proximal right coronary artery to the PA. Surgical ligation of the CAF without coronary bypass was arranged. Intraoperative TEE was successfully employed to localize the CAF, monitor fistula blood flow and heart wall motion, and confirm successful ligation. The patient recovered without complications. This case highlights the utility of intraoperative TEE during ligation of CAF.

## Background

Coronary artery fistulae (CAF) are an uncommon form of coronary anomaly that is typically congenital in origin [1,2]. Angiographic studies have revealed that the incidence of CAF ranges from 0.3 to 0.8% [3]. Clinically, CAF has various manifestations, including myocardial ischemia, stroke, endocarditis, secondary valvular heart disease, and sudden cardiac death [3].

Coronary artery fistulae are typically diagnosed by coronary angiography; limited case reports have described the use of transesophageal echocardiography (TEE) for diagnosing/localizing CAF and/or as a tool during surgery [4-10]. Herein we describe the case of a woman with 2 CAF, which were not found via transthoracic echocardiography, but were detected using coronary angiography and subsequently ligated under guidance of TEE without coronary bypass. Specifically, TEE was used to monitor CAF blood flow and cardiac wall motion.

## Case Presentation

A 61-year-old woman (weight 60 Kg, height 155 cm) with type 2 diabetes mellitus and end-stage renal disease presented to the emergency room with chest pain that began during hemodialysis. The patient described the pain as being dull and retrosternal, without radiation to the shoulder or back. Cold sweating and mild dyspnea on exertion were described. No significant heart murmur was detected. Electrocardiography revealed ST depression in leads V4 to V6. Cardiac enzymes (creatine kinase-MB and troponin I) were within the normal range. The chest x-ray showed widening, elongation, and tortuosity of the thoracic aorta associated with enlargement of the heart. Minimal pulmonary congestion was noted, but no definite abnormal finding was seen in lungs or thoracic cage. The chest x-ray results were in favor of atherosclerotic heart disease and hypertensive cardiovascular heart disease.

The patient was admitted with a tentative diagnosis of unstable angina. After hospitalization, TTE (transthoracic echocardiogram) revealed no abnormalities. Subsequent coronary angiography revealed mild atherosclerosis in the left anterior descending (LAD), left circumflex and right coronary arteries, a large left main coronary artery, and 2 CAF. One CAF extended from the LAD to the pulmonary artery (PA), whereas the other extended from the proximal right coronary artery (RCA) to the PA. Emergent surgical ligation of the CAF without cardiopulmonary bypass was scheduled.

**Figure 1:**
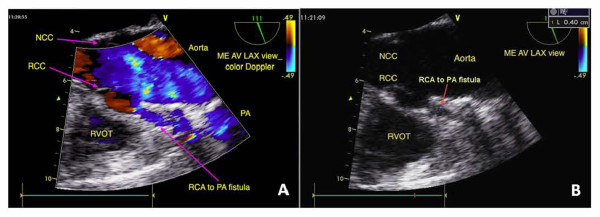
**From the ME AV LAX view, right coronary cusp of aortic valve can be easily figured while RCA is seldom visualized (A)**. However, the abnormal RCA-to-PA fistula of this patient was observed (diameter = 0.4 cm) because of the proximity of the fistula to the RCA origin (B). Mid-esophageal aortic valve long axis-view. NCC: noncoronary cusp, RCC: right coronary cusp, RVOT: Right ventricular outflow tract, RCA: right coronary artery, PA: pulmonary artery.

**Figure 2:**
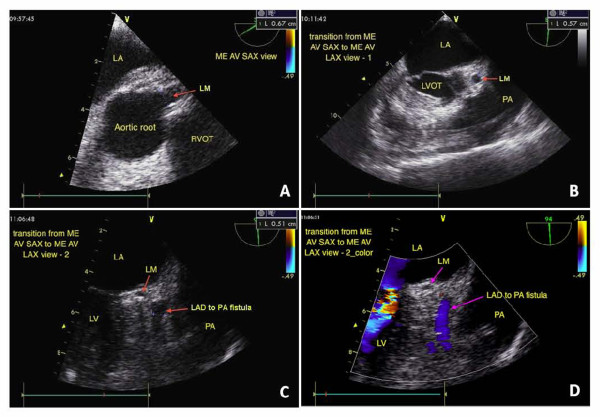
**From the ME AV SAX view, the orifice of LAD-to-PA fistula was not found in the beginning**. The LAD artery to PA fistula (diameter = 0.5 cm) was partially visualized (A); after tracing down to the LM with the view transition from SAX to LAX, the LAD-to-PA fistula was fully visualized with 0.5 cm in diameter by tracing the vessel during transition from the ME AV SAX view to the ME AV LAX view (B, C, D). Mid-esophageal aortic valve short-axis view, LA: left atrium, LM: left main coronary, LV: left ventricle, LVOT: left ventricular outflow tract.

Surgery was performed under general anesthesia. The 2 CAF were identified using TEE and blood flow was recorded before surgical separation of the fistulae. As shown in Figure 1, the proximal RCA to PA fistula (diameter = 0.4 cm) was visualized on the midesophageal aortic valve long-axis (ME AV LAX) view. The LAD artery to PA fistula (diameter = 0.5 cm) was partially visualized on the ME AV short-axis (SAX) view (Figure 2A), and fully visualized by tracing the vessel during transition from the ME AV SAX view to the ME AV LAX view (Figure 2B-D). Three additional videos show these findings in more detail (Additional file 1, Additional file 2, Additional file 3).

After separating the fistulae from adjacent tissue, the fistulae were partially ligated. Blood flow in both fistulae was decreased as observed by TEE color Doppler monitoring. Over the following 10 minutes, no heart wall motion abnormalities were detected as determined by TEE upon ME 4 chamber, ME 2 chamber, and transgastric SAX views. In addition, there were no changes in the ST-T wave pattern or arrhythmia as determined by electrocardiograph monitoring. The fistulae were completely ligated and cessation of fistulae blood flow was confirmed by TEE.

After surgery, the patient recovered in the surgical intensive care unit for 3 days with medication of dopamine at 3 mcg/kg/hr. She received hemodialysis during intensive care and was later transferred to the general ward. After 9 days of hospitalization, the patient was discharged without chest tightness. Cardiac enzyme levels were normal. The patient was followed-up 3 times over the ensuing 3 months after surgery and reported no complications or further chest tightness during hemodialysis.

## Conclusions

Approximately 50% of CAF originate from the RCA, with the most frequent sites of termination being the right atrium or right ventricle [11]. In the present case, 2 left-to-right shunt fistulae were detected, originating from the LAD artery and proximal RCA to the PA. Of note, the CAF in this case were initially identified using coronary angiography after transthoracic echocardiography, a technique which has limitations for the diagnosis of large coronary artery fistulae [12], did not reveal any abnormalities. Prompt surgical intervention was arranged because of the size of the fistulae and the detection of shunts.

A few previous reports have described the use of TEE for diagnosing CAF or as an intraoperative monitoring tool before cardiopulmonary bypass and after ligation [5,8-11]. Several reports have also described the applicability of intraoperative TEE for confirming the cessation of fistula blood flow after ligation [6,13,14]. In the present case, we successfully employed intraoperative TEE to aid in the localization of the target fistulae, monitor fistula blood flow during surgical ligation, and monitor heart wall motion.

A comparison of our results with those in the recent transcatheter and surgical literature shows similar early effectiveness, morbidity and mortality [15]. But the transcatheter approach is a fairly complicated intervention and requires an experienced operator and interventional specialist with expertise in both coronary arteriography and embolization techniques. In our hospital, there was no such specialist who could perform transcatheter embolization. Therefore the patient was transferred to the surgical department for an operation.

Our case highlights the observation that different TEE views can be used to identify different coronary structures and to localize CAF. The ME AV LAX view allows for delineation of structures near the aortic root, including the right coronary cusp of the aortic valve. Although the RCA is seldom evident on this view, we were able to trace the RCA to PA fistula on this view in the present case because of the proximity of the fistula to the RCA origin. The ME AV SAX view allows for delineation of all 3 aortic valve cusps, the left main (LM) coronary artery, the right ventricular outflow tract, and the proximal PA. Upon initial examination, we were not able to locate the orifice of the LAD artery to PA fistula by tracing the LM coronary artery. This fistula was subsequently located upon examination of the transition from the ME AV SAX view to the ME AV LAX view and confirmed by tracing back to the LM coronary artery.

The cause of the woman's chest pain was probably that the fistulae had produced the phenomenon of "coronary steal" [16], that is, by diverting coronary blood flow, the fistulae had reduced blood flow and produced myocardial ischemia in the area distal to the site of the fistula.

Surgical ligation of CAF has a risk of mortality. Indeed, findings from a previous report suggest that this procedure is associated with a mortality rate of 2 to 4%, and a perioperative myocardial infarction rate of 3.6% [4]. Use of intraoperative TEE may reduce the risk of mortality and morbidity associated with this procedure for several reasons. First, CAF can be localized much more rapidly using intraoperative TEE compared with surgical isolation alone, thus shortening the duration of surgery. Second, TEE facilitates selection of the appropriate vessel for ligation, thus reducing the risk of inadvertent iatrogenic injury. Third, TEE allows for continuous intraoperative monitoring, so that abnormalities in cardiac wall motion are readily detected. Finally, TEE can be used to confirm cessation of fistula blood flow, i.e., surgical success.

This case demonstrates the value of using multiple techniques in the diagnosis and surgical treatment of CAF. We were able to diagnose CAF using coronary angiography and employed intraoperative TEE to ensure a safe and successful surgical CAF ligation by monitoring both CAF blood flow and cardiac wall motion.

## Consent

Written informed consent was obtained from the patient for publication of this Case report and any accompanying images. A copy of the written consent is available for review by the Editor-in-Chief of this journal.

## Competing interests

The authors declare that they have no competing interests.

## Supplementary Material

Additional file 1 **Movie 1**. A dynamic display of the echo finding in Figure 1. From the ME AV LAX view, right coronary cusp of aortic valve is easily figured while RCA is seldom visualized. Click here for file

Additional file 2 **Movie 2A**. A dynamic display of the echo findings in Figure 2. From the ME AV SAX view, the orifice of LAD-to-PA fistula was not found in the beginning. Click here for file

Additional file 3 **Movie 2B**. After tracing down to the LM with the view transition from SAX to LAX, the LAD-to-PA fistula was fully visualized. Click here for file
